# Evidence for significant influence of host immunity on changes in differential blood count during malaria

**DOI:** 10.1186/1475-2875-13-155

**Published:** 2014-04-23

**Authors:** Nicole Berens-Riha, Inge Kroidl, Mirjam Schunk, Martin Alberer, Marcus Beissner, Michael Pritsch, Arne Kroidl, Günter Fröschl, Ingrid Hanus, Gisela Bretzel, Frank von Sonnenburg, Hans Dieter Nothdurft, Thomas Löscher, Karl-Heinz Herbinger

**Affiliations:** 1 Department of Infectious Diseases and Tropical Medicine, Medical Centre of the University of Munich, Ludwig-Maximilians-Universität (LMU), Leopoldstraße 5, 80802 Munich, Germany

**Keywords:** Malaria, Differential blood count, Semi-immunity, Monocyte-to-lymphocyte-ratio, Neutrophil-to-lymphocyte-ratio

## Abstract

**Background:**

Malaria has been shown to change blood counts. Recently, a few studies have investigated the alteration of the peripheral blood monocyte-to-lymphocyte count ratio (MLCR) and the neutrophil-to-lymphocyte count ratio (NLCR) during infection with *Plasmodium falciparum*. Based on these findings this study investigates the predictive values of blood count alterations during malaria across different sub-populations.

**Methods:**

Cases and controls admitted to the Department of Infectious Diseases and Tropical Medicine from January 2000 through December 2010 were included in this comparative analysis. Blood count values and other variables at admission controlled for age, gender and immune status were statistically investigated.

**Results:**

The study population comprised 210 malaria patients, infected with *P. falciparum* (68%), *Plasmodium vivax* (21%)*, Plasmodium ovale* (7%) and *Plasmodium malariae* (4%), and 210 controls. A positive correlation of parasite density with NLCR and neutrophil counts, and a negative correlation of parasite density with thrombocyte, leucocyte and lymphocyte counts were found. An interaction with semi-immunity was observed; ratios were significantly different in semi-immune compared to non-immune patients (*P* <0.001).

The MLCR discriminated best between malaria cases and controls (AUC = 0.691; AUC = 0.741 in non-immune travellers), whereas the NLCR better predicted severe malaria, especially in semi-immune patients (AUC = 0.788).

**Conclusion:**

Malaria causes typical but non-specific alterations of the differential blood count. The predictive value of the ratios was fair but limited. However, these changes were less pronounced in patients with semi-immunity. The ratios might constitute easily applicable surrogate biomarkers for immunity.

## Background

In recent years, several publications in different fields have focused on changes in the differential blood count during infectious and other diseases. The ratio of neutrophil-to-lymphocyte count (NLCR) was proposed as a parameter of systemic inflammation and stress in severely ill patients in intensive care unit [1]. De Jager *et al.* concluded that lymphocytopaenia and a high NLCR were better predictors of sepsis than routine parameters like C-reactive protein (CRP) level, white blood cell count and neutrophil count in adults in an emergency care setting [2]. The NLCR was also investigated as a prognostic factor for clinical outcomes in malaria [3].

Thrombocytopaenia, as well as anaemia, are well-known characteristics occurring during and following plasmodial infection [4,5]. Leucocytes were assumed to be rather stable, and in low to normal range in malaria [6,7]. Wolfswinkel *et al.* found that the NLCR correlated with parasite density (parasitaemia) in imported cases. NLCR and lymphocytopaenia were investigated as prognostic markers for treatment outcome of severe malaria cases but both were inferior to CRP levels [3].

The monocyte-to-lymphocyte count ratio (MLCR) was introduced by Warimwe *et al.* (formerly “ML-ratio”) as a parameter for the risk of clinical manifestation of malaria. They concluded that the MLCR could reflect an individual’s protection capacity against clinical manifestation of *Plasmodium falciparum* malaria [8].

In this study, blood count, NLCR, MLCR, neutrophil-to-monocyte count ratio (NMCR) and other unspecific parameters of malaria cases at admission were investigated and compared to those of healthy controls. Results were controlled for age, gender, immune status, and parasitaemia.

## Methods

### Cases and controls

A database from the Department of Infectious Diseases and Tropical Medicine (DITM) of the Ludwig-Maximilians-Universität of Munich recording all clinical, epidemiological and laboratory data of patients who presented from January 2000 through December 2010 was used as source for this analysis. The first group (further referred to as “cases”) comprised all laboratory-confirmed malaria patients of both gender and all age groups. Patients with positive bacterial blood cultures, severe underlying diseases and other concomitant diseases were excluded. Individuals matched by gender and age comprised the control group, further referred to as “controls”. A control was defined as a healthy individual without acute or severe underlying chronic diseases at admission who had been admitted for a certificate of fitness before working in tropical regions.

Gender, age, origin correlating with immunity against malaria, country of travel, blood count including differentiation, and lactate dehydrogenase (LDH) were included in the main analyses. Liver enzymes (alanine aminotransferase aspartate transaminase, gamma-glutamyltransferase), bilirubin and creatinine were used in subanalyses for malaria patients only. Dependent of their birthplace in endemic malaria areas according to the WHO [9], study participants were categorized as non-immune (NI) or semi-immune (SI) travellers.

### Ethical considerations

All cases and controls provided written informed consent that their biological samples and that consequent results may be used for research purposes and publication in a fully anonymized manner. Ethical clearance was approved by the Ethical Committee of the Ludwig-Maximilians-Universität.

### Laboratory diagnostics

All laboratory procedures were performed as part of routine diagnostics. Thin and thick blood smears were prepared by specialized laboratory technicians and stained by Romanowski stain (Diff-Quick®) and 10% Giemsa solution for 20 min, respectively. All smears were independently read by two experienced microscopists. Two-hundred fields of thick smears were read, thin smears were used for species discrimination and quantification of parasitaemia expressed in per cent. Blood count was measured with an automated system (KX-21 N, Sysmex®) and differential blood count was read manually by microscopy. LDH and other liver enzymes were analysed by an external, accredited laboratory.

### Database and statistics

Data were de-identified and transferred from medical report forms into an electronic database by double entry and cleaned by two independent researchers. The homogeneity of both groups was based on age and gender. The first null hypothesis stated that there is no difference of the MLCR in cases compared to controls. Likewise the second null hypothesis was defined with the NLCR as outcome variable. The null hypotheses were assessed by comparing the median ratios in each group using the non-parametric, unpaired, two-sample Mann-Whitney-Wilcoxon test, as no normal distribution was assumed. For further analysis, ratios were categorized and tested in a logistic regression model controlled for gender, age and variables significantly associated with the outcome affirmed by likelihood ratio test. Predictive accuracy was determined by the calculation of the area under the ROC (receiver operating characteristics) curve (AUC). Correlations between continuous variables were analysed by linear regression models if appropriate. All variables were tested for interaction. The statistical software used was Stata, version 11.0 (StataCorp, Texas, USA).

## Results

### Baseline data

The univariate analysis of baseline data is presented in Additional file 1. Two third were infected with *P. falciparum*, 21% with *Plasmodium vivax*, 7% with *Plasmodium ovale*, and 4% with *Plasmodium malariae*. Only two mixed infections with *P. falciparum* and *P. vivax* were included in the analysis and counted as *P. falciparum*; parasitaemia was below 1%.

Median age of all travellers was 36 years. Matching of very old cases with controls was difficult as controls were recruited from the working population (*P* =0.005). Most plasmodial infections were acquired in West Africa (60%), followed by East, Central and Southern Africa (19%), Asia with Pacific region (19%), and Latin America (2%). Additional file 2 shows the distribution of the four species by region.

The proportion of SI travellers in the control group was negligible (2.4%) but one third of the cases were categorized as SI. *Plasmodium falciparum* was found in 91 and 58% of SI and NI cases, respectively, whereas *P. vivax* was more common in NI (30%) than SI cases (3%). Ninety per cent of SI travellers returned from East Africa. Patients returning from East Africa were half SI and half NI travellers. In all other regions, SI cases were under-represented. Parasitaemia ranged from 0.1 to 13% in *P. falciparum*; nine patients had a parasitaemia ≥2%. All other species showed a parasitaemia ≤1%. Parasitaemia in NI cases was similar to densities in SI cases (*P* = 0.224). Thirty-three *P. falciparum* and five *P. vivax/Plasmodium ovale* patients showed signs and symptoms of severe malaria at admission including hyperparasitaemia (≥2%), 3-times elevated liver enzymes and bilirubin ≥ 3.0 mg/dL[10,11]. Median LDH was significantly elevated in cases compared to controls and above the normal range. None of the patients was admitted with renal failure or cerebral symptoms; all patients recovered. Malaria in SI patients was generally more benign than in NI patients.

**Figure 1:**
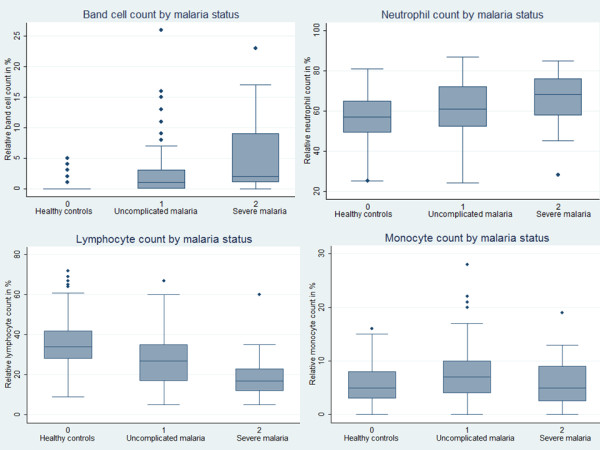
**White blood cell count by malaria status.** The graphs show the median as well as the 25^th^ and 75^th^ percentiles and adjacent values (range). Upper line, left: The median band cell count was significantly higher in malaria patients compared to controls (*P* <0.001) as well as in severe malaria patients compared to uncomplicated cases (*P* <0.001). Upper line, right: The median neutrophil count was significantly higher in malaria patients compared to controls (*P* <0.001) as well as in severe malaria patients compared to uncomplicated cases (*P* <0.001). Lower line, left: The median lymphocyte count was significantly lower in malaria patients compared to controls (*P* <0.001) as well as in severe malaria patients compared to uncomplicated cases (*P* <0.001). Lower line, right: The median monocyte count in malaria patients was only slightly but significantly higher than in controls (*P* <0.001), whereas the median monocyte count in severe malaria patients was significantly decreased than in uncomplicated cases (*P* <0.001) and similar to controls (*P* = 0.729).

Median haemoglobin, thrombocyte count, overall leucocyte as well as lymphocyte count were significantly lower in cases (Figure 1). Median lymphocyte and thrombocyte count were significantly lower in severe than in uncomplicated malaria (*P* <0.001 each).

**Figure 2:**
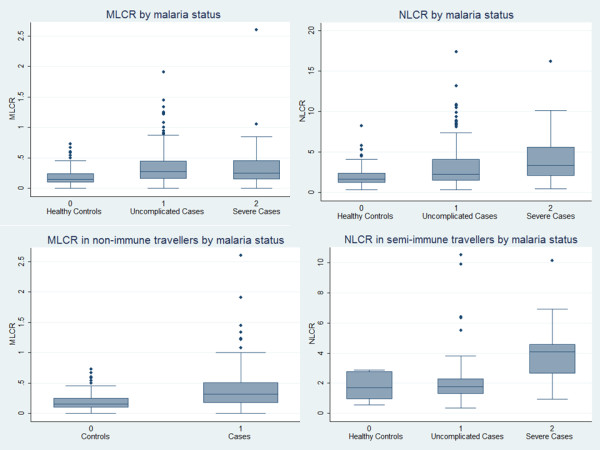
**MLCR and NLCR by malaria and immune status.** The graphs show the median as well as the 25^th^ and 75^th^ percentiles and adjacent values (range). Upper line, left: The median MLCR in malaria patients was significantly higher than in controls (*P* <0.001), while it was similar in severe malaria patients and uncomplicated cases (*P* =0.631). Upper line, right: The median NLCR was significantly higher in malaria patients compared to controls (*P* <0.001) as well as in severe malaria patients compared to uncomplicated cases (*P* =0.012). Lower line, left: The median MLCR in NI malaria patients was significantly higher than in NI controls (*P* <0.001). The difference in SI patients was not significant. Lower line, right: The median NLCR in SI severe cases was significantly higher than in SI uncomplicated cases (*P* =0.003). The difference was less pronounced in NI cases.

Mean absolute and relative monocytes, neutrophils and bands were significantly higher in malaria patients than in controls but within the normal range (Figure 1). In consequence, the MLCR and NLCR were significantly higher in all malaria patients than in controls (Figure 2). The evident difference could be shown for all species except for *Plasmodium malariae*, most probably due to the very low sample size (Additional file 1).

### Linear and logistic regression models for continuous variables

All correlations if not otherwise mentioned were controlled for age, gender and immune status. A significant positive correlation between parasitaemia and absolute or relative neutrophil as well as band cell count in all cases was shown (*P* <0.001 each). Relative and absolute lymphocyte counts were negatively correlated with parasitaemia (NI: *P* <0.001 each; SI: *P* =0.009 and *P* =0.024, respectively). The relation of monocytes with parasitaemia was non-linear. Monocyte count was elevated in uncomplicated malaria but decreased in severe malaria, which was observed in SI and NI cases. In consequence, no evident correlation of parasitaemia with the MLCR or NMCR was found but a strong positive correlation with the NLCR (*P* = 0.014) could be shown for all cases (Additional file 3). The risk for severe malaria and hyperparasitaemia (≥2%) was positively correlated with the NLCR (*P* =0.023 and *P* =0.057, respectively). Also thrombocytopaenia showed a strong positive correlation with parasitaemia.

### MLCR, NLCR, and NMCR stratified by gender, age, and immune status

Median monocyte count was significantly lower in healthy women compared to healthy men. In consequence, healthy women generally had a slightly but significantly lower MLCR and higher NMCR than men (*P* =0.004 and *P* =0.019, respectively). The difference was not detectable in acutely ill patients. Median neutrophil and lymphocyte count, and the NLCR were similar in both genders.

There was no interaction with age detected. In children ≤15, no differences of the ratios between cases and controls were observed but the analysis was hampered due to the very low sample size.

**Figure 3:**
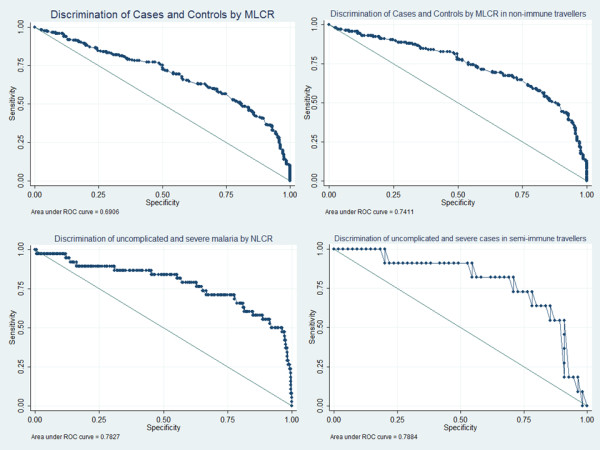
**Area under the ROC curve.** Upper line, left: Discrimination of cases and controls by MLCR. The area under the receiver operating characteristics (ROC) curve (AUC) is 0.691. Upper line, right: Discrimination of cases and controls in non-immune travellers by MLCR. The AUC is 0.741. Lower line, left: Discrimination of uncomplicated and severe cases by NLCR. The AUC is 0.783. Upper line, right: Discrimination of uncomplicated and severe cases in semi-immune travellers by NLCR. The AUC is 0.788.

An interaction with semi-immunity was observed. The significant difference between controls and cases shown in the NI population was not detectable in SI cases (Table 1). The MLCR and NLCR in SI cases were significantly lower than in NI cases. Both ratios of SI and NI controls showed no significant differences but sample size of SI controls was very low. Discrimination of NI cases and controls by MLCR showed a fair accuracy (Figure 3).

**Table 1:** MLCR and NLCR in different variables

	**Median MLCR (IQR)**			**Median NLCR (IQR)**		
	**Malaria negative**	**Malaria positive**	**p-value**	**Malaria negative**	**Malaria positive**	**p-value**
Semi-immune	0.10 (0.07; 0.21)	0.20 (0.10; 0.35)	.185^§^	1.69 (0.77; 2.83)	1.86 (1.31; 3.19)	.529^§^
Non-immune	0.16 (0.10; 0.25)	0.32 (0.17; 0.52)	**<.001**	1.70 (1.20; 2.40)	2.88 (1.66; 5.19)	**<.001**
Female	0.13 (0.06; 0.22)	0.26 (0.17; 0.37)	**<.001**	1.72 (1.18; 2.34)	2.48 (1.36; 4.78)	**.002**
Male	0.17 (0.11; 0.26)	0.29 (0.15; 0.48)	**<.001**	1.66 (1.20; 2.41)	2.46 (1.61; 4.47)	**<.001**
Uncomplicated	-	0.28 (0.15; 0.45)		-	2.23 (1.50; 4.09)	
Severe	-	0.25 (0.14; 0.47)	.631	-	3.62 (2.09; 5.69)	**.012**
Age groups (years)						
1-6	0.09 (0.06; 0.14)	0.16 (0.10; 0.85)	.127^§^	1.74 (0.82; 1.74)	1.00 (0.81; 1.06)	.275^§^
7-15	0.21 (0.16; 0.24)	0.17 (0.12; 0.23)	.462^§^	1.07 (0.96; 1.97)	1.20 (0.76; 5.96)	.917^§^
16-30	0.15 (0.09; 0.24)	0.30 (0.17; 0.52)	**<.001**	1.74 (1.15; 2.27)	2.28 (1.38; 3.98)	**.008**
31-45	0.16 (0.11; 0.26)	0.25 (0.13; 0.38)	**.004**	1.78 (1.18; 2.61)	2.49 (1.51; 4.32)	**.005**
46-60	0.17 (0.10; 0.24)	0.29 (0.15; 0.48)	**<.001**	1.66 (1.22; 2.27)	3.38 (2.10; 5.62)	**<.001**
61-79	0.16 (0.04; 0.32)	0.42 (0.20; 0.54)	**.008**	1.59 (1.44; 2.50)	3.61 (1.96; 5.90)	**.004**

^§^Very low sample size.

Medians (with 25 and 75% percentiles/IQR) of MLCR and NLCR are given. Ranked means were compared by Mann-Wilcoxon-Whitney test and p-values were presented. P-values <0.05 are marked in bold.

Due to the characteristics of the monocytes, the NMCR was significantly decreased in NI cases compared to controls and significantly elevated in severe malaria compared to uncomplicated malaria (Additional file 4).

Cell counts showed no significant differences between cases and controls in SI travellers but comparison of severe and uncomplicated SI cases revealed a significantly lower median in relative monocyte count as well as significantly lower lymphocyte and higher neutrophil counts in severe cases (Table 2). The median NLCR was significantly elevated in severe cases compared to uncomplicated cases (*P* =0.003, Figure 2). Discrimination between severe and uncomplicated malaria by NLCR in SI cases showed fair accuracy (Figure 3).

**Table 2:** MLCR, NLCR, NMCR, and different variables stratified by immune status

	**Semi-immune patients**			**Non-immune patients**		
	**Malaria negative N = 5**	**Malaria positive N = 66**	**p-value**	**Malaria negative N = 205**	**Malaria positive N = 144**	**p-value**
Female, n (%)	4 (80)	21 (32)		59 (29)	44 (31)	
Male, n (%)	1 (20)	45 (68)	**.030**	146 (71)	100 (69)	.882
Leucocytes /μL	5,900 (4,950; 7,700)	4,900 (3,900; 5,950)	.096	6,000 (5,000; 7,000)	4,850 (3,925; 6,000)	**<.001**
Monocytes in%,	5 (3; 6)	6 (3; 9)	.315	6 (3; 8)	7 (4; 10)	**<.001**
/μL	276 (162; 373)	274 (141; 436)	.745	315 (196; 485)	340 (208; 503)	.513
Lymphocytes in%,	36 (24; 54)	32 (22; 38)	.405	34 (27; 41)	22 (14; 32)	**<.001**
/μL	2,124 ( 1,576; 3,190)	1,325 (989; 2,041)	.059	1,971 (1,563; 2,415)	1,081 (722; 1,542)	**<.001**
Neutrophils in%,	61 (40; 67)	59 (50; 66)	.857	57 (49; 65)	65 (53; 74)	**<.001**
/μL	3,082 (2,324; 4,737)	2,880 (1,862; 3,642)	.406	3,306 (2,648; 4,248)	3,006 (2,184; 4,007)	**.019**
MLCR	0.10 (0.07; 0.21)	0.20 (0.10; 0.32)	.185	0.16 (0.08; 0.22)	0.32 (0.17; 0.52)	**<.001**
NLCR	1.69 (0.77; 2.83)	1.86 (1.31; 3.19)	.574	1.70 (1.20; 2.40)	2.88 (1.66; 5.19)	**<.001**
NMCR	11.17 (7.43; 38.75)	9.63 (6.31; 19.33)	.473	10.27 (6.88; 16.81)	8.33 (5.45; 15.90)	**.031**
Thrombocytes*1000	239 (142; 270)	116 (82; 183)	**.024**	229 (190; 262)	117 (84; 153)	**<.001**
/μL						
	**Uncomplicated malaria, 55/66 (83)**	**Severe malaria 11/66 (17)**		**Uncomplicated malaria, 117/144 (81)**	**Severe malaria 27/144 (19)**	
Female, n (%)	17 (31)	4 (36)		35 (30)	9 (33)	
Male, n (%)	38 (69)	7 (64)	.723	82 (70)	18 (67)	.728
Leucocytes /μL	4,900 (3,900; 5,950)	5,800 (4,600; 6,300)	.068	4,700 (3,850; 5,900)	5,400 (4,000; 6,000)	.249
Monocytes in%,	6 (3; 9)	3 (2; 5)	**.027**	7 (5; 10)	7 (3; 9)	.170
/μL	274 (141; 436)	210 (106; 245)	.126	354 (215; 497)	280 (156; 540)	.364
Lymphocytes in%,	32 (22; 38)	18 (15; 29)	**.004**	22 (15; 32)	20 (13; 27)	.213
/μL	1,325 (989; 2,041)	1,044 (828; 1,827)	**.049**	1,113 (718; 1,534)	936 (754; 1,560)	.582
Neutrophils in%,	59 (50; 66)	68 (61; 78)	**.006**	63 (53; 75)	68 (52; 73)	.732
/μL	2,880 (1,862; 3,642)	3,588 (3,339; 4,914)	**.005**	3,000 (2,170; 3,944)	3,120 (2,250; 4,144)	.519
MLCR	0.20 (0.10; 0.35)	0.25 (0.09; 0.31)	.925	0.32 (0.19; 0.49)	0.31 (0.15; 0.54)	.526
NLCR	1.86 (1.31; 3.19)	3.83 (2.10; 4.59)	**.003**	2.76 (1.64; 4.84)	3.40 (2.04; 5.92)	.279
NMCR	8.86 (5.78; 13.33)	23.33 (11.89; 33.38)	**.019**	8.29 (5.36; 15.00)	9.30 (6.14; 24.08)	.193
Thrombocytes (1000/μL)	116 (82; 183)	92 (59; 111)	**.022**	124 (93; 163)	100 (51; 118)	**.003**

Medians (with 25 and 75% percentiles/IQR) of are given if not otherwise mentioned. Ranked means were compared by Mann-Wilcoxon-Whitney test and p-values were presented. P-values <0.05 are marked in bold.

*times.

### Null hypothesis MLCR and NLCR

To categorize the MLCRs in NI and SI travellers, the lower limit of the 95% CI in malaria cases in each group was chosen as cut-off (Table 3). Multivariate analysis with logistic regression models adjusted for gender and age showed a six times higher risk for malaria when the MLCR was ≥0.27 in NI travellers (95% CI 3.7-9.6; *P* <0.001). Sensitivity, specificity and the positive predictive value (PPV) for malaria were 59.0, 80.0, and 67.5%, respectively. As SI malaria cases had a significantly lower MLCR, an accordingly lower cut-off was chosen; the lower limit of the 95% CI in malaria patients was 0.15. However, no evident difference between controls and cases was found (P = 0.200).

**Table 3:** Null hypothesis tested by logistic regression: categorized MLCR and NLCR controlled for age and gender, interaction by immunity against malaria

**MLCR**								
Non-immune				Semi-immune				
	All N (%)	MLCR ≥ 0.27	Odds ratio (95% CI)	p-value	All N (%)	MLCR ≥ 0.15	Odds ratio (95% CI)	p-value
Malaria negative	205 (58.7)	41/205 (20.0)	base	**<.001**	5 (7.0)	2/5 (40.0)	base	.200
Malaria positive	144 (41.3)	85/144 (59.0)	**5.92 (3.66-9.59)**		66 (93.0)	42/66 (63.6)	3.70 (0.50-27.43)	
**NLCR**								
Non-immune				Semi-immune				
	All N (%)	NLCR ≥ 2.5	Odds ratio (95% CI)	p-value	All N (%)	NLCR ≥ 1.5	Odds ratio (95% CI)	p-value
Malaria negative	205 (58.7)	43/205 (21.0)	base	**<.001**	5 (7.0)	3/5 (60.0)	base	.983
Malaria positive	144 (41.3)	83/144 (57.6)	**5.24 (3.25-8.45)**		66 (93.0)	39/66 (59.1)	0.98 (0.13-7.18)	

P-values <0.05 are marked in bold.

Similar results could be shown for the NLCR using logistic regression models. A 5.2-times higher risk for malaria was found in NI travellers but no difference for SI travellers (Table 3). However, sensitivity and specificity of NLCR for severe malaria in SI cases were 63.6 and 80.0%, respectively. The prevalence-dependent PPV was only 38.8%.

## Discussion

As described in other studies [3-7], blood cell counts revealed significant differences in malaria patients compared to controls. As expected, thrombocytes were significantly reduced in plasmodial infections as described elsewhere [4]. Leucocyte counts were decreased or in normal range in malaria patients as previously reported [6,7]. There was a negative correlation with parasite density and leukocythaemia. A similar correlation was found with septic disease, and could be interpreted as marker of stress [2].

The majority of patients with imported *P. falciparum* malaria [12] as well as in endemic areas [13-16] had lymphocytopaenia. This could be confirmed. Median lymphocyte counts were significantly higher in SI than in NI patients but were similar in both control groups. In summary, malaria induces lymphocytopaenia, and this trend is more pronounced in NI patients.

The association of lymphocyte count and severity of malaria has been controversially reported [1,3,17]. In this study, parasitaemia and lymphocyte count were negatively correlated. In NI patients, hyperparasitaemia of ≥2% is a defined sign of severe malaria. However, this criterion has to be used with caution for SI patients from holo-endemic countries [10]. Interestingly, there was no significant difference of lymphocytes between severe and uncomplicated NI cases but between severe and uncomplicated SI cases (0.004).

Lymphocytopaenia is reported to be accompanied by an increase in neutrophil count. This is interpreted as a sign of systemic inflammation and stress, and occurs in many infectious and non-infectious diseases [14,15,18].

In a recent study, the efficacy of the RTS,S vaccine, measured by malaria episodes during follow-up, was negatively correlated with the MLCR at vaccination [19]. The higher MLCR might be due to submicroscopic parasitaemia or other acute infections during vaccination that could impair the outcome. Independently of the individual vaccine efficacy, underlying infections potentially resulting in an elevated MLCR at the time of vaccination in combination with a very low efficacy raises once again the general question about the outcome of vaccinations in case of acute or chronic diseases during vaccination. Markers like the MLCR might be able to predict vaccine failures.

The differences in neutrophil count and NLCR between malaria patients and controls were distinct (Table 1). Wolfwinkel *et al*. found a positive correlation of NLCR and parasitaemia in patients with imported malaria [3]. Median NLCR of their cases was 3.2 compared to 2.5 (2.9 in NI patients) in the present study. A significant rise in total leucocyte and lymphocyte counts accompanied by a significant decrease in NLCR was observed after parasite clearance [3]. As normalization generally happens after successful recovery, the healthy controls constitute an adequate means of comparison for changes during malaria, although intra-individual analysis before, during and after disease should be more accurate.

The linear correlation of parasitaemia with lymphocytes or neutrophils was not detectable for monocytes (Figure 1). Similar results were shown by Chiwakata *et al.*; they propagated a protective effect in malaria by high levels of inducible nitric oxide synthase mRNA that is associated with an increased monocyte count in uncomplicated cases. Patients with severe malaria presented with a low to normal monocyte count, similar to those of controls [20].

The distinct difference of relative white blood cell count and both ratios depending on the immune status against malaria was the most interesting finding in this study. However, data have to be interpreted with caution. Definition of semi-immunity is difficult, especially when accurate data on the exact origin, living conditions, disease episodes in the past, the duration of parasite-free intervals, and antibody titres are missing. This might result in an overestimation of semi-immunity in this group. Semi-immune patients in the NI group seem to be rather unlikely. The similar ratios and differential blood count constellations in all severe cases independent of the theoretical immune status raise the question whether those 11 severe SI patients might have lost immunity. In any way, the striking difference in uncomplicated cases remains.

The MLCR was measured by Warimwe *et al.* as parameter for the risk of clinical manifestation of malaria in children [8]. Asymptomatic parasitaemia at survey was associated with less clinical episodes during follow-up compared to individuals without parasitaemia at the time of inclusion. The MLCR was positively correlated with the frequency of clinical episodes in patients that carried an asymptomatic parasitaemia at recruitment. One might hypothetically conclude that the higher the MLCR during asymptomatic parasitaemia, the lower the degree of immunity and the lower the immunological ability to keep the parasites on a subclinical level. The MLCR might be helpful during clinical trials to further discriminate different levels of semi-immunity.

The most critical part of the study is the low sample size of healthy SI travellers. All references to this group might not be reliable as shown normal blood values might not be representative. The non-significant lower MLCR in SI controls could be related to the high female proportion (80%) as healthy women in general had a significantly lower MLCR than male controls (*P* =0.004). The number of children under 15 years was very limited, accurate analysis was impossible but should be performed with a bigger sample size to investigate leucocyte behaviour in children with malaria.

A recent study from Tanzania presented laboratory reference values for healthy adults [21]. Blood counts differed slightly in Tanzanian individuals resulting in a theoretical median NLCR of 1.19. Data on monocytes were not presented. The difference between median NLCR of ill and healthy NI individuals was 1.18 (2.88-1.70) but 0.17 (1.86-1.69) in SI persons in the present study. Using the theoretical median NLCR from Tanzania as a baseline for SI controls, the difference was 0.67. It might be significant though it is almost 50% less than in NI individuals.

## Conclusion

The NLCR correlated well with parasitaemia and severity of infection in NI and SI cases. This could serve as a critical parameter, especially for severe malaria, and should be evaluated with cerebral malaria patients. The MLCR discriminated fairly between malaria patients and controls in NI travellers but showed significantly lower values in uncomplicated SI patients. This might correlate to acquired immunity against malaria and could help to discriminate different levels of immunity. Further investigation of these surrogate markers in symptomatic and asymptomatic SI patients might reveal the mechanisms behind these changes based on immune-systemic adaption.

## Competing interests

The authors declare that they have no competing interests.

## Authors’ contributions

NBR, IK, MS, MA, MB, MP, AK, GF, and IH were responsible for the clinical work. GB was responsible for the laboratory supervision. NBR and KH were responsible for data management and statistical analysis. NBR, FS, HDN, and TL designed the study. NBR wrote the first draft of the paper. All authors contributed to the interpretation of the data and to writing the manuscript. All authors read and approved the final manuscript.

## Supplementary Material

Additional file 1 Baseline data. Univariate analysis of all variables. Click here for file

Additional file 2 **Distribution of imported malaria cases by country of acquisition.***Plasmodium falciparum* was mainly acquired in West Africa (77%), *P. vivax* predominantly in Asia and the Pacific Region (70%), *P. ovale* mostly in Africa (82%) and *P. malariae* (75%) mainly in Africa. Almost all *P. vivax* cases from Africa were imported from Ethiopia. Click here for file

Additional file 3 **Correlation of NLCR and parasitaemia.** NLCR and parasitaemia were positively correlated. The correlation fitted best with low to moderate parasitaemia. The sample size of cases with high parasitaemia (≥2%) was low. Click here for file

Additional file 4 **NMCR in different variables.** Medians (with 25 and 75% percentiles/IQR) of the NMCR are given. Ranked means were compared by Mann-Wilcoxon-Whitney test and p-values were presented. P-values <0.05 are marked in bold. There is no linearity of monocytes. NMCR behaves contrary to MLCR. There is no difference between severe and uncomplicated by MLCR but between cases and controls. There is only a slight difference between uncomplicated cases and controls by NMCR (the difference lacks significance if the sample size is small, i.e. different age groups) but a distinct difference between severe and uncomplicated cases. Discrimination of severe und uncomplicated cases was better by NLCR than NMCR. Click here for file

## References

[B1] Zahorec R Ratio of neutrophil to lymphocyte counts - rapid and simple parameter of systemic inflammation and stress in critically ill Bratisl Lek Listy 2001 102 5 14 11723675

[B2] de Jager CP van Wijk PT Mathoera RB de Jongh-Leuvenink J van der Poll T Wever PC Lymphocytopenia and neutrophil-lymphocyte count ratio predict bacteremia better than conventional infection markers in an emergency care unit Crit Care 2010 14 R192 10.1186/cc9309 21034463 PMC3219299

[B3] van Wolfswinkel ME Vliegenthart-Jongbloed K de Mendonça Melo M Wever PC McCall MB Koelewijn R van Hellemond JJ van Genderen PJ Predictive value of lymphocytopenia and the neutrophil-lymphocyte count ratio for severe imported malaria Malar J 2013 12 101 10.1186/1475-2875-12-101 23506136 PMC3608093

[B4] Herbinger KH Schunk M Nothdurft HD von Sonnenburg F Löscher T Bretzel G Comparative study on infection-induced thrombocytopenia among returned travellers Infection 2012 40 373 379 10.1007/s15010-012-0242-9 22350868

[B5] Herbinger KH Metzner M Schmidt V Beissner M Nothdurft HD von Sonnenburg F Löscher T Infection-induced anaemia: a cross-sectional study of 14,636 German travellers aged 20–49 years Infection 2013 41 1079 1087 10.1007/s15010-013-0528-6 24014235

[B6] McKenzie FE Prudhomme WA Magill AJ Forney JR Permpanich B Lucas C Gasser RA Jr Wongsrichanalai C White blood cell counts and malaria J Infect Dis 2005 192 323 330 10.1086/431152 15962228 PMC2481386

[B7] Akinosoglou KS Solomou EE Gogos CA Malaria: a haematological disease Hematology 2012 17 106 114 10.1179/102453312X13221316477336 22664049

[B8] Warimwe GM Murungi LM Kamuyu G Nyangweso GM Wambua J Naranbhai V Fletcher HA Hill AV Bejon P Osier FH Marsh K The ratio of monocytes to lymphocytes in peripheral blood correlates with increased susceptibility to clinical malaria in Kenyan children PLoS One 2013 8 e57320 10.1371/journal.pone.0057320 23437368 PMC3577721

[B9] WHO World Malaria Report: 2013 2013 Geneva: World Health Organization [http://www.who.int/malaria/publications/world_malaria_report_2013/report/en/]

[B10] WHO Guidelines for Malaria Treatment, 2010 2010 2 Geneva: World Health Organization [http://whqlibdoc.who.int/publications/2010/9789241547925_eng.pdf]

[B11] World Health Organization Severe falciparum malaria Trans R Soc Trop Med Hyg 2000 94 S1 1 90 11103309

[B12] Richards MW Behrens RH Doherty JF Hematologic changes in acute, imported *Plasmodium falciparum* malaria Am J Trop Med Hyg 1998 59 859 9886188 10.4269/ajtmh.1998.59.859

[B13] Lisse IM Aaby P Whittle H Knudsen K A community study of 3 T lymphocyte subsets and malaria parasitaemia Trans R Soc Trop Med Hyg 1994 88 709 710 10.1016/0035-9203(94)90242-9 7886782

[B14] Wyllie DH Bowler IC Peto TE Relation between lymphopenia and bacteraemia in UK adults with medical emergencies J Clin Pathol 2004 57 950 955 10.1136/jcp.2004.017335 15333656 PMC1770434

[B15] Hawkins CA Collignon P Adams DN Bowden FJ Cook MC Profound lymphopenia and bacteraemia Intern Med J 2006 36 385 388 10.1111/j.1445-5994.2006.01076.x 16732866

[B16] Kassa D Petros B Mesele T Hailu E Wolday D Characterization of peripheral blood lymphocyte subsets in patients with acute *Plasmodium falciparum* and *P. vivax* malaria infections at Wonji Sugar Estate, Ethiopia Clin Vaccine Immunol 2006 13 376 379 10.1128/CVI.13.3.376-379.2006 16522780 PMC1391951

[B17] Ladhani S Lowe B Cole AO Kowuondo K Newton CR Changes in white blood cells and platelets in children with falciparum malaria: relationship to disease outcome Br J Haematol 2002 119 839 847 10.1046/j.1365-2141.2002.03904.x 12437669

[B18] Seebach JD Morant R Ruegg R Seifert B Fehr J The diagnostic value of the neutrophil left shift in predicting inflammatory and infectious disease Am J Clin Pathol 1997 107 582 591 9128272 10.1093/ajcp/107.5.582

[B19] Warimwe GM Fletcher HA Olotu A Agnandji ST Hill AV Marsh K Bejon P Peripheral blood monocyte-to-lymphocyte ratio at study enrollment predicts efficacy of the RTS.S malaria vaccine: analysis of pooled phase II clinical trial data BMC Med 2013 1 184 23962071 10.1186/1741-7015-11-184 PMC3765422

[B20] Chiwakata CB Hemmer CJ Dietrich M High levels of inducible nitric oxide synthase mRNA are associated with increased monocyte counts in blood and have a beneficial role in *Plasmodium falciparum* Infect Immun 2000 68 394 399 10.1128/IAI.68.1.394-399.2000 10603415 PMC97148

[B21] Saathoff E Schneider P Kleinfeldt V Geis S Haule D Maboko L Samky E de Souza M Robb M Hoelscher M Laboratory reference values for healthy adults from southern Tanzania Trop Med Int Health 2008 13 612 625 10.1111/j.1365-3156.2008.02047.x 18331386

