# Supplementary material for: Evidence for significant influence of host immunity on changes in differential blood count during malaria
Source: Malar J. 2014 Apr 23;13:155. doi: 10.1186/1475-2875-13-155 (PMC4021259; doi:10.1186/1475-2875-13-155)
Supplement: Additional file 1 — Baseline data. Univariate analysis of all variables. [file 1475-2875-13-155-S1.docx]

**Additional File 1. Baseline data**

If not otherwise defined, median (with 25% and 75% percentiles/IQR) is given. P-values <0.05 are marked in bold.

|  | **All** | **Controls** | **Malaria** | **p-value** | ***falciparum*** | ***vivax*** | ***ovale*** | ***malariae*** |
| --- | --- | --- | --- | --- | --- | --- | --- | --- |
| N  (%) | 420 | 210/420  (50.0) | 210/420  (50.0) |  | 143/210  (68.1) | 45/210  (21.4) | 14/210  (6.7) | 8/210  (3.8) |
| Female  (%) | 128/420  (30.5) | 63/210  (30.0) | 65/210  (31.0) | .832^1^ | 46/143  (32.2) | 14/45  (31.1) | 5/14  (35.7) | 0/8  (0) |
| (%) | (30.5) | (30.0) | (31.0) | .832^1^ | (32.2) | (31.1) | (35.7) | (0) |
| Median Age | 36 (28; 49) | 36 (29; 49) | 35 (27; 49) | .536^2^ | 35 (27; 46) | 33 (26; 51) | 36 (26; 67) | 33.5 (32; 43) |
| Age groups (years) | n/420 (%) | n/210 (%) | n/210 (%) |  | n/143 (%) | n/45 (%) | n/14 (%) | n/8 (%) |
| 1-6 | 6 (1.4) | 3 (1.4) | 3 (1.4) | 1^2^ | 3 (2.1) | 0 | 0 | 0 |
| 7-15 | 10 (2.4) | 5 (2.4) | 5 (2.4) | .371^2^ | 4 (2.8) | 1 (2.2) | 0 | 0 |
| 16-30 | 119 (28.3) | 57 (27.1) | 62 (29.5) | .856^2^ | 41 (28.7) | 15 (33.3) | 5 (35.7) | 1 (12.5) |
| 31-45 | 160 (38.1) | 80 (38.1) | 80 (38.1) | 1^2^ | 57 (39.9) | 14 (31.1) | 3 (21.4) | 6 (75.0) |
| 46-60 | 92 (21.9) | 49 (23.3) | 43 (20.5) | .512^2^ | 28 (19.6) | 12 (26.7) | 2 (14.3) | 1 (12.5) |
| 61-79 | 33 (7.9) | 16 (7.6) | 17 (8.1) | **.005^2^** | 10 (7.0) | 3 (6.7) | 4 (28.6) | 0 |
| Non-immune, n (%) | 349 (83.1) | 205 (97.6)^3^ | 144 (68.6) | **<.001^1^** | 83 (58.0) | 43 (95.6) | 12 (85.7) | 6 (75.0) |
| Semi-immune, n (%) | 71 (16.9) | 5 (2.4)^3^ | 66 (31.4) |  | 60 (42.0) | 2 (4.4) | 2 (14.3) | 2 (25.0) |
| Uncompl. mal., n (%) | - | - | 172 (81.9) |  | 110 (76.9) | 40 (88.9) | 14 (100) | 8 (100) |
| Severe malaria, n (%) | - | - | 38 (18.1) |  | 33 (23.1) | 5 (11.1) | 0 | 0 |
| Parasitaemia, in % | - | - | 1 (0.1; 1) |  | 1 (0.1; 1) | 1 (1; 1) | 1 (1;1) | 1 (0.3; 1) |
| Haemoglobin  in mg/dl | 14.1  (12.1; 15.9) | 14.4  (12.8; 16.1) | 13.6  (11.9;15.7) | **<.001^2^** | 13.6^4^  (11.7; 16.0) | 13.6^4^  (12.2; 14.4) | 12.8^4^  (11.6; 13.5) | 13.5  (12.6; 14.1) |
| Leucocytes/µL | 5400  (4400; 6700) | 6000  (5000; 7000) | 4900  (3900; 6000) | **<.001^2^** | 4800^4^  (3900; 6000) | 4700^4^  (4000; 5700) | 5600  (4675; 6050) | 4550^4^  (3400; 6600) |
| Thrombocytes  *1,000/µL | 180  (114; 233) | 229  (189; 262) | 115.5  (83; 162) | **<.001^2^** | 118^4^  (80; 168) | 102^4^  (85; 136) | 153^4^  (107; 175) | 125^4^  (102; 162) |
| Lymphocytes  in /µL | 30 (21; 38),  1641  (1092; 2173) | 34 (27; 41),  1998  (1565; 2419) | 25 (16; 34),  1157  (798; 1708) | **<.001^2^**  **<.001^2^** | 24 (16; 35)^4^,  1147^4^  (756; 1872) | 26 (15; 33)^4^,  1120^4^  (852; 1518) | 26 (17; 34)^4^,  1359^4^  (1074; 1834) | 29 (19-36),  1300^4^  (951; 1346) |
| Monocytes  in %, in /µL | 6 (4; 9),  306  (192; 468) | 6 (3; 8),  311  (196; 482) | 7 (4; 9),  304  (190; 464) | **<.001^2^**  .418 | 7 (3; 9)^4^,  285  (185; 448) | 7 (4; 12)^4^,  376  (189; 550) | 8 (5; 11) ^4^,  381  (294; 526) | 7 (4;11)^4^,  314  (164; 519) |
| Neutrophils  in %, in /µL | 59 (51; 68)  3124  (2420; 4140) | 57 (49; 65)  3303  (2645; 4236) | 62 (53; 73)  2967  (2146; 3932) | **<.001^2^**  **<.018^2^** | 63 (52; 73)^4^,  2920  (2124; 3843) | 59 (53; 73)^4^,  3016  (2146; 3930) | 62 (56; 75),  3625  (4171; 2498) | 62 (53; 69)  2936  (1734; 4507) |
| Band cells  in %, in /µL | 0 (0; 1)  0 (0; 66) | 0 (0; 0)  0 (0; 0) | 1 (0; 3)  49 (0; 160) | **<.001^2^**  **<.001^2^** | 1 (0; 3) ^4^,  46 (0; 162) ^4^ | 1 (0; 4) ^4^,  61 (0; 162) ^4^ | 1 (0; 2) ^4^,  61 (0; 114) ^4^ | 1 (0; 3) ^4^,  36 (0; 164) ^4^ |
| MLCR | 0.20  (0.12; 0.33) | 0.16  (0.10; 0.25) | 0.28  (0.15; 0.45) | **<.001^2^** | 0.26^4^  (0.15; 0.44) | 0.29^4^  (0.15; 0.51) | 0.29^4^  (0.19; 0.42) | 0.30  (0.12; 0.35) |
| NLCR | 1.98  (1.35; 3.22) | 1.70  (1.19; 2.41) | 2.47  (1.56; 4.55) | **<.001^2^** | 2.52^4^  (1.51; 4.59) | 2.28^4^  (1.60; 4.72) | 2.31^4^  (1.61; 4.16) | 2.13  (1.56; 4.00) |
| NMCR | 9.71 | 10.33 | 8.79 | **0.046** | 8.39 | 7.41^4^ | 7.39 | 8.67 |
|  | (6.25; 16.50) | (6.88; 16.75) | (5.61; 16.28) |  | (6.11; 17.3) | (5.09; 14.44) | (5.49; 13.00) | (4.91; 21.00) |

^1^ χ^2^-test.

^2^ Unpaired two-sample Mann-Whitney-Wilcoxon test, if appropriate.

^3^ Supposed semi-immunity: born in malaria-endemic country. Controls from non-endemic countries were compared to controls from endemic countries.

^4^ Each malaria group compared to controls by unpaired two-sample Mann-Whitney-Wilcoxon test: *P* < .010. Low sample size for *P. malariae*.
