# Supplementary figures and images for: Evidence for significant influence of host immunity on changes in differential blood count during malaria
Source: Malar J. 2014 Apr 23;13:155. doi: 10.1186/1475-2875-13-155 (PMC4021259; doi:10.1186/1475-2875-13-155)

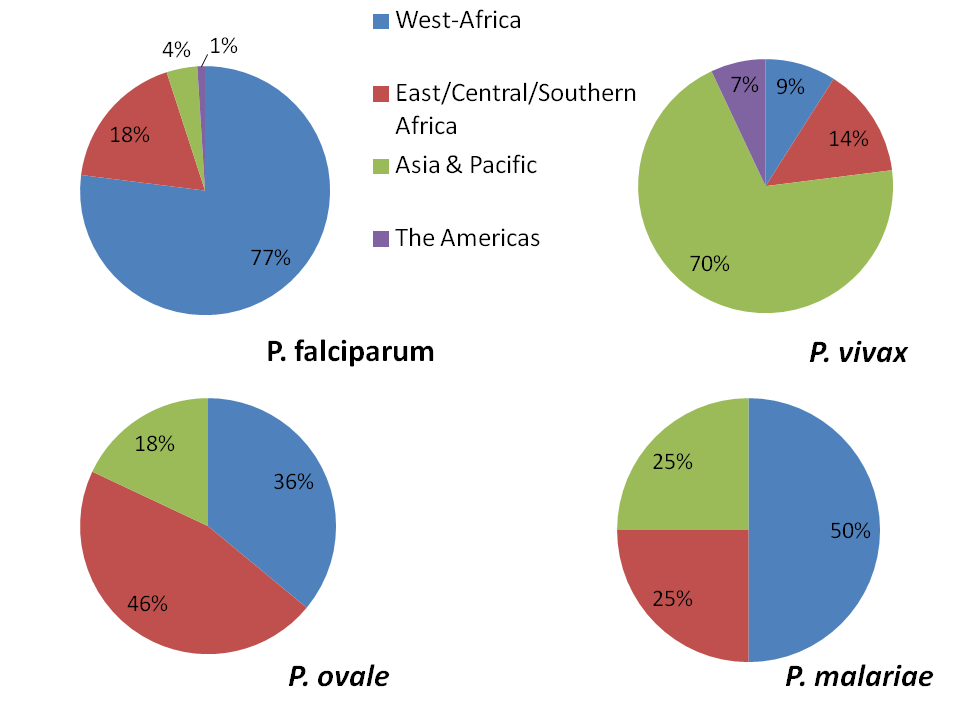

Supplement: Additional file 2 — Distribution of imported malaria cases by country of acquisition.Plasmodium falciparum was mainly acquired in West Africa (77%), P. vivax predominantly in Asia and the Pacific Region (70%), P. ovale mostly in Africa (82%) and P. malariae (75%) mainly in Africa. Almost all P. vivax cases from Africa were imported from Ethiopia. [file 1475-2875-13-155-S2.tiff]

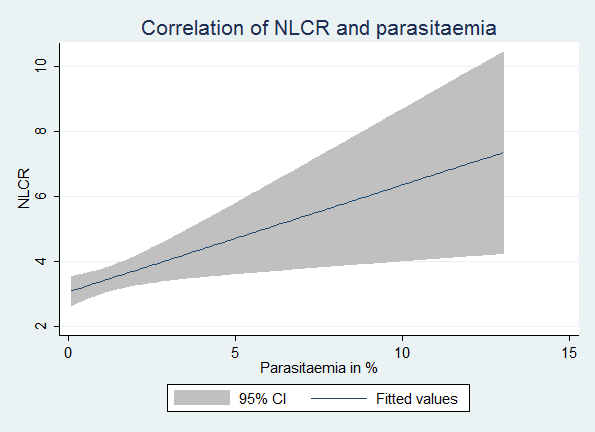

Supplement: Additional file 3 — Correlation of NLCR and parasitaemia. NLCR and parasitaemia were positively correlated. The correlation fitted best with low to moderate parasitaemia. The sample size of cases with high parasitaemia (≥2%) was low. [file 1475-2875-13-155-S3.tiff]
