# Supplementary material for: Evidence for significant influence of host immunity on changes in differential blood count during malaria
Source: Malar J. 2014 Apr 23;13:155. doi: 10.1186/1475-2875-13-155 (PMC4021259; doi:10.1186/1475-2875-13-155)
Supplement: Additional file 4 — NMCR in different variables. Medians (with 25 and 75% percentiles/IQR) of the NMCR are given. Ranked means were compared by Mann-Wilcoxon-Whitney test and p-values were presented. P-values <0.05 are marked in bold. There is no linearity of monocytes. NMCR behaves contrary to MLCR. There is no difference between severe and uncomplicated by MLCR but between cases and controls. There is only a slight difference between uncomplicated cases and controls by NMCR (the difference lacks significance if the sample size is small, i.e. different age groups) but a distinct difference between severe and uncomplicated cases. Discrimination of severe und uncomplicated cases was better by NLCR than NMCR. [file 1475-2875-13-155-S4.docx]

**Additional file 4. NMCR in different variables**

Medians (with 25 and 75% percentiles/IQR) of NMCR are given. Ranked means were compared by Mann-Wilcoxon-Whitney test and p-values were presented. P-values <0.05 are marked in bold.

|  | | Median NMCR (IQR) | | |
| --- | --- | --- | --- | --- |
|  | | Malaria negative | Malaria positive | p-value |
| Semi-immune | | 11.17 (7.43; 38.75) | 9.63 (6.31; 19.33) | .473 |
| Non-immune | | 10.27 (6.88; 16.81) | 8.33 (5.45; 15.90) | **.031** |
| Female | | 12.20 (7.29; 24.33) | 8.25 (6.13; 17.50) | **.033** |
| Male | | 9.77 (6.69; 15.00) | 9.00 (5.14; 16.35) | .351 |
| Uncomplicated | | - | 8.46 (5.41; 15.85) |  |
| Severe | | - | 13.60 (6.31; 25.75) | **.022** |
| Age groups (years) | | |  |  |
| 1-6 | 19.67 (5.71; 32.00) | | 6.14 (1.25; 8.4) | .275 |
| 7-15 | 6.88 (5.06; 9.24) | | 8.25 (6.29; 24.46) | .465 |
| 16-30 | 10.71 (6.81; 17.25) | | 8.71 (6.34; 13.87) | .103 |
| 31-45 | 10.75 (7.69; 15.33) | | 9.67 (5.10; 17.85) | .237 |
| 46-60 | 10.33 (6.38; 16.63) | | 10.60 (6.78; 19.00) | .833 |
| 61-79 | 9.17 (4.91; 34.00) | | 6.80 (5.09; 17.64) | .333 |
